# Riboflow: Using Deep Learning to Classify Riboswitches With ∼99% Accuracy

**DOI:** 10.3389/fbioe.2020.00808

**Published:** 2020-07-14

**Authors:** Keshav Aditya R. Premkumar, Ramit Bharanikumar, Ashok Palaniappan

**Affiliations:** ^1^MS Program in Computer Science, Department of Computer Science, College of Engineering and Applied Sciences, Stony Brook University, Stony Brook, NY, United States; ^2^MS in Bioinformatics, Georgia Institute of Technology, Atlanta, GA, United States; ^3^Department of Bioinformatics, School of Chemical and Biotechnology, SASTRA Deemed University, Thanjavur, India

**Keywords:** riboswitch family, synthetic biology, machine learning, convolutional neural network, recurrent neural network, hyperparameter optimization, multiclass ROC, clustering

## Abstract

Riboswitches are *cis-*regulatory genetic elements that use an aptamer to control gene expression. Specificity to cognate ligand and diversity of such ligands have expanded the functional repertoire of riboswitches to mediate mounting apt responses to sudden metabolic demands and signal changes in environmental conditions. Given their critical role in microbial life, riboswitch characterisation remains a challenging computational problem. Here we have addressed the issue with advanced deep learning frameworks, namely convolutional neural networks (CNN), and bidirectional recurrent neural networks (RNN) with Long Short-Term Memory (LSTM). Using a comprehensive dataset of 32 ligand classes and a stratified train-validate-test approach, we demonstrated the accurate performance of both the deep learning models (CNN and RNN) relative to conventional hyperparameter-optimized machine learning classifiers on all key performance metrics, including the ROC curve analysis. In particular, the bidirectional LSTM RNN emerged as the best-performing learning method for identifying the ligand-specificity of riboswitches with an accuracy >0.99 and macro-averaged F-score of 0.96. An additional attraction is that the deep learning models do not require prior feature engineering. A dynamic update functionality is built into the models to factor for the constant discovery of new riboswitches, and extend the predictive modeling to new classes. Our work would enable the design of genetic circuits with custom-tuned riboswitch aptamers that would effect precise translational control in synthetic biology. The associated software is available as an open-source Python package and standalone resource for use in genome annotation, synthetic biology, and biotechnology workflows.

## Availability:

PyPi package: riboflow @ https://pypi.org/project/riboflow

Repository with Standalone suite of tools: https://github.com/RiboswitchClassifier

Language: Python 3.6 with numpy, keras, and tensorflow libraries.

License: MIT.

## Introduction

Riboswitches are ubiquitous and critical metabolite-sensing gene expression regulators in bacteria that are capable of folding into at least two alternative conformations of 5′UTR mRNA secondary structure, which functionally switch gene expression between on and off states (Mandal et al., 2003; Roth and Breaker, 2009; Serganov and Nudler, 2013). The selection of conformation is dictated by the binding of ligand cognate to the aptamer domain of a given riboswitch (Gelfand et al., 1999; Winkler et al., 2002, 2004). Cognate ligands are key metabolites that mediate responses to metabolic or external stimuli. Consequent to conformational changes, riboswitches weaken transcriptional termination or occlude the ribosome binding site thereby inhibiting translation initiation of associated genes (Yanofsky, 1981; Mandal and Breaker, 2004). Riboswitches provide an intriguing window into the ‘RNA world’ biology (Stormo and Ji, 2001; Brantl, 2004; Breaker et al., 2006; Strobel and Cochrane, 2007) and there is evidence of their wider distribution in complex genomes (Sudarsan et al., 2003; Barrick and Breaker, 2007; Bocobza and Aharoni, 2014; McCown et al., 2017). The modular properties of riboswitches have engendered the possibility of synthetic control of gene expression (Tucker and Breaker, 2005), and combined with the ability to engineer binding to an *ad hoc* ligand, riboswitches have turned out to be a valuable addition to the synthetic biologist’s toolkit (Wieland and Hartig, 2008; Wittmann and Suess, 2012). In addition to orthogonal gene control they are useful in a variety of applications, notably metabolic engineering (Zhou and Zeng, 2015), biosensor design (Yang et al., 2013; Meyer et al., 2015) and genetic electronics (Villa et al., 2018). Riboswitches have been used as basic computing units of a complex biocomputation network, where the concentration of the ligand of interest is titrated into measurable gene expression (Beisel and Smolke, 2009; Domin et al., 2017). Riboswitches have also been directly used as posttranscriptional and translational checkpoints in genetic circuits (Chang et al., 2012). Their key functional roles in infectious agents but absence in host genomes make them attractive targets for the design of cognate inhibitors (Blount and Breaker, 2006; Deigan and Ferré-D’Amaré, 2011; Wang et al., 2017). Characterisation of riboswitches would expand the repertoire of translational control options in synthetic biology and bioengineering. In turn, this would facilitate the reliable construction of precise genetic circuits. In view of their myriad applications, robust computational methods for the accurate characterisation of novel riboswitch sequences would be of great value.

Since the discovery of riboswitches (Mironov et al., 2002; Nahvi et al., 2002), many computational efforts have been advanced for their characterisation, notably Infernal (Nawrocki and Eddy, 2013), Riboswitch finder (Bengert and Dandekar, 2004), RibEx (Abreu-Goodger and Merino, 2005), RiboSW (Chang et al., 2009) and DRD (Havill et al., 2014), and reviewed in Clote (2015) and Antunes et al. (2017). These methods used probabilistic models of known classes with or without secondary structure information to infer or predict the riboswitch class. Singh and Singh explored featuring mono-nucleotide and di-nucleotide frequencies in a supervised machine learning framework to classify different riboswitch sequences, and concluded that the multi-layer perceptron was optimal (Singh and Singh, 2016). Their work achieved modest performance (F-score of 0.35 on 16 different riboswitch classes). None of the above methods were shown to generalize effectively to unseen riboswitches. Our remedy was to explore the use of deep learning models for riboswitch classification. Deep networks are relatively recent neural network-based frameworks that use a type of learning known as representation learning (Bengio et al., 2013). Convolutional neural networks are one type of deep learning, known for hierarchical information extraction. Such architectures with alternating convolutional and pooling layers have been earlier used to extract structural and functional information from genome sequences (Alipanahi et al., 2015; Sønderby et al., 2015; Zhou and Troyanskaya, 2015; Kelley et al., 2016). Recurrent neural networks are counterparts to CNNs and specialize in extracting features from time-series data (Che et al., 2018). RNNs with Long Short-Term Memory (termed LSTM) incorporate recurrent connections to model long-run dependencies in sequential information (Hochreiter and Schmidhuber, 1997), such as in speech and video (Graves and Schmidhuber, 2005). This feature of LSTM RNNs immediately suggests their use in character-level modeling of biological sequence data (Lipton, 2015; Lo Bosco and Di Gangi, 2017). Bidirectional LSTM RNN have been shown to be especially effective, given that they combine the outputs of two LSTM RNNs, one processing the sequence from left to right, the other one from right to left, together enabling the capture of dynamic temporal or spatial behavior (Sundermeyer et al., 2014). Bidirectional LSTM RNNs are a particularly powerful abstraction for modeling nucleic acid sequences whose spatial secondary structure determines function (Lee et al., 2015). Two recent successes of deep learning methods in RNA biology have been: (i) prediction of RNA secondary structure (Singh et al., 2019), and (ii) dynamic range improvement in riboswitch devices (Groher et al., 2019). Here we have evaluated the relative merits of a spectrum of state-of-the-art learning methods for resolving the ligand-specificity of riboswitches from sequence. It is demonstrated that the deep learning models vastly outperformed other machine learning models with respect to the classification of riboswitches belonging to 32 different families.

## Materials and Methods

### Dataset and Pre-processing

We searched the Rfam database of RNA families (Kalvari et al., 2018) with the term “Riboswitch AND family” and the corresponding hit sequences were obtained in FASTA format from the Rfam ftp server (Rfam v13 accessed on July 6, 2019). Each riboswitch is represented by the coding strand sequence, with uracil replaced by thymine, thereby conforming to the nucleotide alphabet ‘ACGT.’ Each sequence was scanned for non-standard letters (i.e., other than the alphabet) and such occurrences were corrected using the character mapping defined in Table 1. The feature vectors for machine learning were extracted from the sequences. For each sequence, 20 features were computed, comprising four mononucleotide frequencies (A,C,G,T) and 16 dinucleotide frequencies. To address possible skew in distribution, all the frequency features were normalized to zero mean and unit variance. Deep models, namely convolutional neural networks (CNNs) and bidirectional recurrent neural networks with LSTM (hereafter simply referred as RNNs) are capable of using the sequences directly as the feature space, obviating any need for feature engineering. We used the first 250 bases of the riboswitch sequence as the input, with the proviso that shorter sequences (which is usually the case; Table 2) were padded for the extra spaces. Python scripts used to create the final dataset are available in the repository for this project^1^. The dataset consists of the riboswitch sequence, four 1-mer frequencies, 16 2-mer frequencies, and class, for each instance, which could be appropriately subsetted for training the base and deep models.

**TABLE 1:** Non-standard nucleotide mapping.

S. No.	Original letter	Mapped character	^#^Occurrences in dataset
1	R	G	6
2	Y	T	8
3	K	G	1
4	S	G	3
5	W	A	2
6	H	A	2

*Rare occurrences of non-standard nucleotides in the sequences were converted using this mapping key.*

**TABLE 2:** A summary of the riboswitch dataset used in our study.

Class no.	Rfam ID	Class Name	Class size	Avg. length
1	RF00504	Glycine riboswitch	4592	100
2	RF01786	Cyclic di-GMP-II riboswitch	661	86
3	RF01750	ZMP/ZTP riboswitch	1674	92
4	RF00059	Thiamine pyrophosphate riboswitch	12559	110
5	RF01057	SAH Riboswitch	832	92
6	RF01725	SAM -1 -4 Variant riboswitch	793	104
7	RF00162	SAM - 1 Riboswitch	6027	113
8	RF00174	Cobalamin riboswitch	14212	189
9	RF01055	Molybdenum Co-factor riboswitch	1221	134
10	RF01727	SAM-SAH Riboswitch	240	50
11	RF01482	AdoCbl riboswitch	182	137
12	RF03057	nhaA-I RNA	559	56
13	RF01734	Fluoride Riboswitch	2018	70
14	RF00167	Purine Riboswitch	2632	101
15	RF00234	Glucosamine-6-phosphate riboswitch	936	175
16	RF01739	Glutamine riboswitch	1103	64
17	RF03072	raiA RNA	736	219
18	RF03058	sul1 RNA	344	56
19	RF00380	Ykok riboswitch (Magnesium sensing)	1059	170
20	RF00168	Lysine Riboswitch	2240	180
21	RF03071	DUF1646	265	53
22	RF01689	AdoCbl variant RNA	212	125
23	RF00379	ydaO/yuaA leader	3918	164
24	RF00634	SAM - 4 Riboswitch	1245	116
25	RF01767	SAM - 3 Riboswitch	195	90
26	RF00080	yybP-ykoY manganese riboswitch	833	158
27	RF02683	NiCo riboswitch (Nickel or Cobalt sensing)	235	97
28	RF00442	Guanidine-I riboswitch	902	109
29	RF00522	Pre-queosine riboswitch -1	533	45
30	RF00050	Flavin Mononucleotide Riboswitch	4080	142
31	RF01831	THF riboswitch	663	102
32	RF00521	SAM - 2 Riboswitch	819	78

*The dataset includes a mixture of metabolite/ion/cofactor/amino-acid/nucleotide/vitamin/signaling-molecule aptamer ligands. ‘Class no.’ corresponds to the response classes to be learnt in machine learning. The average length of all sequences in a given class is also given.*

### Predictive Modeling

The machine learning problem is simply stated as: given the riboswitch sequence, predict the ligand class of the riboswitch. Toward this, a battery of eight supervised machine learning and deep classifiers were studied and evaluated in the present work (Table 3). Classifiers derived from implementations in the Python scikit-learn machine learning library (Pedregosa et al., 2011)^2^ are referred to as base models and include the Decision Tree, K-nearest Neighbors, Gaussian Naive Bayes, the ensemble classifiers AdaBoost and Random Forest and the Multi-layer Perceptron. The deep classifiers namely CNN and RNN derived from implementations in the Python keras library^3^ on tensorflow (Abadi et al., 2015). Three scripts in the repository, namely baseModels.py, rnnApp.py, and cnnApp.py, implement the base models, RNN, and CNN, respectively. For both the base and deep classifiers, the dataset was split into 0.9:0.1 training:test sets. Multi-class modeling is fraught with overfitting to particular classes (especially pronounced in cases of extreme class skew). To address this issue, two strategies were adopted: (i) the splitting process was stratified on the class, which ensured that each class was proportionately and sufficiently distributed in both the training and test sets, and (ii) hyperparameter optimisation, discussed below.

**TABLE 3:** Description of the base model and deep classifiers.

Classifier	Features used	Hyperparameters of interest	ML Library
Decision Tree	1- and 2-mer frequencies	Maximum features, Minimum sample split, Minimum sample leaf, Random state, Max depth	Sklearn
Gaussian Naïve Bayes	1- and 2-mer frequencies	Priors	Sklearn
k-Nearest Neighbors	1- and 2-mer frequencies	Number of neighbors Leaf size, Weights, Algorithm	Sklearn
Adaptive Boosting	1- and 2-mer frequencies	Number of estimators, Learning rate, Algorithm	Sklearn
Random Forest	1- and 2-mer frequencies	Number of estimators, Max depth, Impurity criterion	Sklearn
Multi-layer Perceptron	1- and 2-mer frequencies	Activation, Solver, Alpha (regularization term), Learning rate, epochs, hidden layers, nodes per hidden layer	Sklearn
CNN	Riboswitch sequence	Number of filters, Kernel size, Activation function, Pooling method, number of Conv1D layers, Dropout ratio, Optimiser, #Epochs	TensorFlow (Keras)
Bidirectional RNN with LSTM	Riboswitch sequence	Activation function, number of LSTM nodes, number of Bidirectional layers, Dropout ratio, Optimiser, #Epochs	TensorFlow (Keras)

*Hyperparameters noted for each classifier are meant to be representative. For the deep models, any long riboswitch genome sequences were truncated to 250 nucleotides, which is an adjustable parameter (max_len) set to much larger than the average length of any riboswitch class. The Python3 library used for implementation of machine learning model is noted.*

### Hyperparameter Optimisation

Hyperparameter fine tuning for each classifier was effected by discrete combinatorial grid search on the hyperparameters associated with that classifier. The grid search was evaluated with 10-fold cross-validation of the training set. This yielded the optimal hyperparameters for each classifier. The scripts for hyperparameter optimisation of the base models are available in the repository. In the case of the deep models, we used a train-validate-test approach to model optimisation with keras/TensorFlow, by setting the ‘validation’ flag to 0.1.

### Evaluation Metrics

The performance of each classifier was evaluated on the test set using the receiver operating characteristic (ROC) analysis in addition to standard metrics such as the precision, recall, accuracy and F-score (harmonic mean of precision and recall) (van Rijsbergen, 1975). The ROC curve was obtained by plotting the TP rate vs. the FP rate, i.e., sensitivity vs. (1 – specificity), and the area under the ROC curve (AUROC) could be estimated to rate the model’s performance. AUROC represents the probability that a given classifier would rank a randomly chosen positive instance higher than a randomly chosen negative one. ROC analysis is robust to class imbalance, typical of the machine learning problem at hand, however, a multi-class adaptation of the binary ROC is necessary. For each classifier, this is achieved by computing classwise binary AUROC values in a one-vs.-all manner, followed by aggregating the classwise AUROC values into a single multi-class AUROC measure (Yang, 1999; Manning et al., 2008). Aggregation of the classwise AUROC values could be done in at least two ways:

1. Micro-average AUROC, where each *instance* is given equal weight.

2. Macro-average AUROC, where each *class* is given equal weight.

In micro-averaging, all the instances from different classes are treated equally, to arrive at the final metrics. In particular, the microaverage AUROC is given by the area under the overall TP rate vs. overall FP rate curve.

On the other hand, the macro-average of a given metric for a multi-class prediction problem is estimated by the average of the metric for the individual classes. For example, the macro-average AUROC is given by:

Macro-averageAUROC=(AUROC+1AUROC+2⋯+AUROC)32/32

where AUROC_*i*_ is the binary AUROC for the i^*th*^ class.

It is clear that the micro-average AUROC would be dominated by the larger classes, while the macro-average AUROC is a more balanced measure. Both the micro-average and macro-average AUROC measures were used to evaluate the performance of all our classifiers. A python script, multiclass.py available in the repository, generates all the performance metrics and plots.

### Dynamic Extension of the Models

Genome sequencing of diverse, exotic prokaryotes is likely to yield new regulatory surprises mediated by riboswitches (Breaker, 2011). A model that could classify a fixed set of 32 classes remains static in the wake of exponentially growing number of known genomes. To address the challenge of extending the model to new classes, we have devised a strategy for dynamically updating the model. This process is initiated by feeding the sequences corresponding to the new class(es) to an updater script, which revises the dataset and then trains a new model. The automation of modeling would ensure a user-friendly pipeline for learning any number of riboswitch classes along with the production of performance metrics of such models. The script dynamic.py, available in the repository, implements the dynamic functionality of our modeling effort.

## Results

Our Rfam query retrieved 39 riboswitch class hits, however, seven of these classes had a membership of less than 100 sequences and were filtered out. Subsequently, our dataset consisted of 32 riboswitch classes with a total of 68,520 sequences. A summary of this dataset is presented in Table 2. The largest classes include the cobalamin and thiamine pyrophosphate classes, with >10,000 riboswitches in each, accounting for considerable diversity within classes. Classes with >4,000 members include Flavin mononucleotide (FMN) and glycine riboswitches. Other notable classes with at least 1,000 members include the lysine, purine, fluoride and glutamine switches. The riboswitch sequences were inspected for the standard alphabet (Table 1) and the final pre-processed comma-separated values (csv) datafile with each instance containing the sequence, 20 features and riboswitch class was prepared (available at^4^).

**FIGURE 1:**
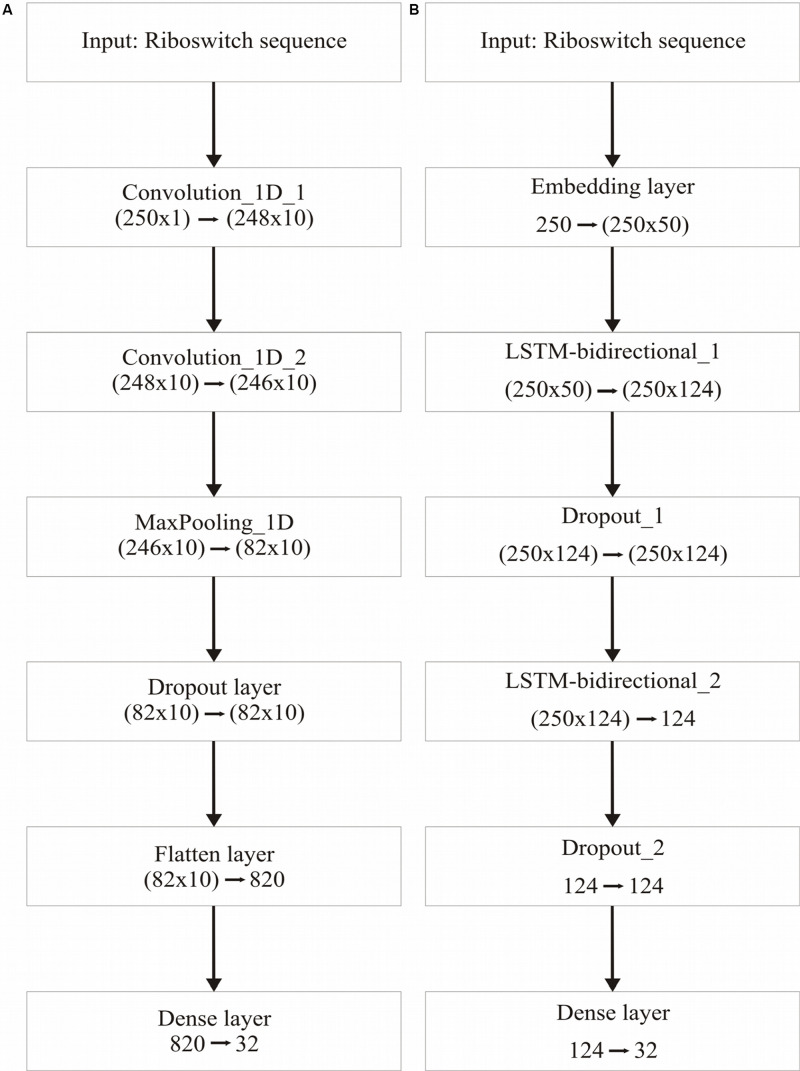
Deep learning frameworks used in the study. **(A)**, CNN architecture, optimized for two 1-dimensional convolutional layers; and **(B)**, Bidirectional RNN with LSTM, optimized for two bidirectional layers. Two dropout layers are used in the RNN.

**FIGURE 2:**
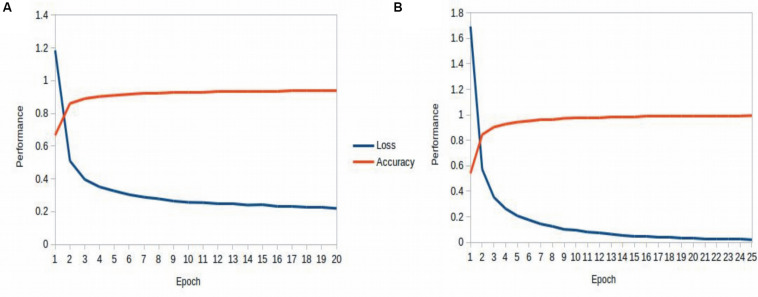
Epoch tuning curves for the CNN **(A)** and RNN **(B)**. The CNN converges faster with respect to the number of epochs, however, the RNN learns better, as seen with the continuously decreasing loss function.

Table 3 recapitulates the key properties of the classifier models used in this study. We noticed poor performance of the base models on the test set with default model parameters, which could be traced to persistent overfitting (dominated by the larger classes), despite stratified sampling. Hyperparameter optimisation of the default parameters is one solution to address this problem and was carried out on the base models. The exercise is summarized in Appendix I (Supplementary File S1), which includes the final configuration of the hyperparameter space for all the base and deep classifiers. The optimized CNN and RNN architectures are illustrated in Figure 1. In the CNN, two convolutional layers were used followed by a pooling layer and dropout layer before flattening to a fully connected output layer. The RNN employed two sophisticated bidirectional LSTM units sandwiched by dropout layers before flattening to a fully connected output layer. The number of training epochs necessary for each deep model was determined based on the convergence of the error function (shown in Figure 2).

**FIGURE 3:**
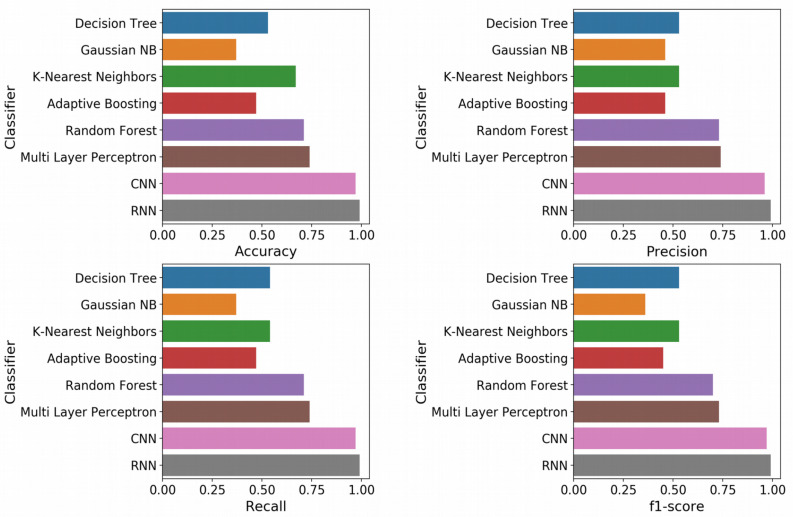
Standard performance metrics. Clockwise from top left, Accuracy; Precision; F-score; and Recall. The overall precision, recall and F-score were computed by macro-averaging the classwise scores. The deep models emerged as vastly superior alternatives to the base machine learning models on all performance metrics.

**FIGURE 4:**
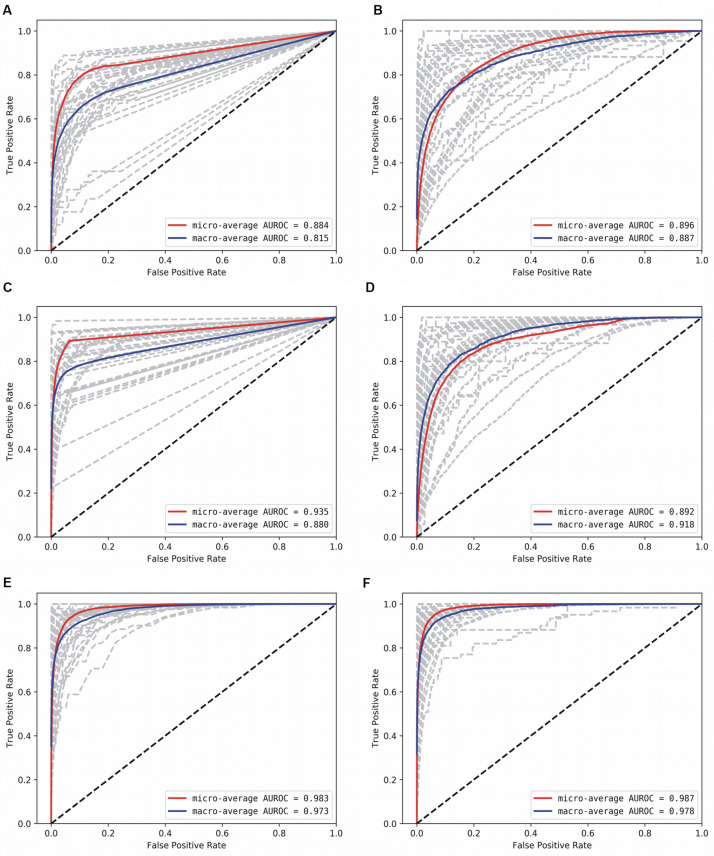
AUROC for the base models. **(A)** Decision Tree, **(B)** Gaussian NB, **(C)** kNN, **D:** AdaBoost, **(E)** Random Forest, and **(F)** Multi-layer Perceptron. Gray lines denote classwise AUROCs of all 32 classes, from which it is clear that not all classes are equally learnt.

**FIGURE 5:**
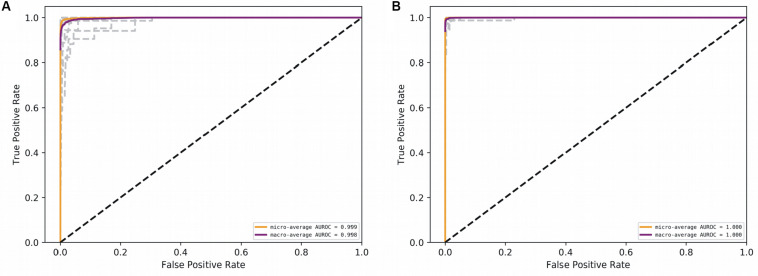
AUROC for the deep models. **(A)** CNN, and **(B)** RNN. Gray lines denote classwise AUROCs of all 32 classes. It clear that RNN achieves learning perfection at both the macro and classwise levels.

With the optimized classifiers, the performance of the predictive modeling was evaluated on the unseen testing set. Figure 3 shows the resultant classifier performance by an array of metrics including accuracy, and F-score. It is abundantly clear that the deep models vastly outperformed the base classifiers in all metrics across all classes. Figures 4, 5 show the ROC curves along with the micro-averaged and macro-averaged AUROC for the base models and the deep models, respectively. The AUROC is indicative of the quality of the overall model. It is seen that the AUROC is 1.00 for all classes for the RNN. Table 4 summarizes the performance of the classifiers, with the detailed classwise F-score of each classifier available in the Supplementary Table S2 and the classwise break-up of the AUROC of all classifiers in the Supplementary Table S3.

**TABLE 4:** Performance metrics for all classifiers.

Model	Accuracy	Precision	Recall	F-score	Micro AUROC	Macro AUROC
Decision Tree	0.54	0.49	0.39	0.42	0.88	0.81
Gaussian NaïveBayes	0.37	0.39	0.46	0.38	0.9	0.89
K-neighbors	0.67	0.75	0.55	0.61	0.94	0.88
AdaBoost	0.47	0.42	0.36	0.36	0.89	0.92
Random Forest	0.71	0.86	0.58	0.65	0.98	0.97
Multi-layer perceptron	0.74	0.75	0.67	0.70	0.99	0.98
CNN	0.97	0.98	0.91	0.93	1	1
RNN	0.99	0.97	0.96	0.96	1	1

*The macro-averaged values of precision, recall and F-score are shown. Micro AUROC, micro-average AUROC; Macro AUROC, macro-average AUROC.*

## Discussion

The RNN model marginally (but clearly) outperformed the CNN model, and both of them significantly outperformed all the base models on all key metrics, notably accuracy and F-score. The best-performing among the base models was the Multi-layer Perceptron. It is noteworthy that the AUROC approached unity and near-perfection for both the deep models, especially the bidirectional RNN with LSTM. This implied that the use of k-mer features masked long-range information whereas the deep models were able to capture such correlations directly from the full sequence. These results affirmed that RNNs could be used to effectively simulate the interactions in biological sequences.

The F-score (a measure of balanced accuracy) is a more unforgiving metric than AUROC in the case of multi-class problems (Table 4). While the CNN and RNN had macro-averaged F-scores of 0.93 and 0.96, respectively, none of the base models exceeded 0.70 including the multilayer perceptron. Supplementary Table S2 provides the classwise F-scores of all classifiers. All the base models struggled to classify the largest riboswitch classes, namely TPP and Cobalamin. This is a consequence of the diversity of such large riboswitch classes, making the ‘outlier’ members harder to classify correctly. Both the RNN and CNN are mapping the sequence input to its corresponding riboswitch family. In such a case, the sequence similarity within a family and sequence dissimilarity across families represent plausible discriminating features that the models are learning. Higher order features such as sequence context and base dependencies dictated by RNA secondary structure also constitute learnable information. Table 5 shows the results of a sequence-based clustering analysis of the riboswitch families using the cd-hit algorithm (Li and Godzik, 2006). It is seen that there are >7000 and >10,000 singleton clusters in the TPP and Cobalamin classes, respectively. Here we introduce a diversity metric for riboswitch families, defined as the ratio of the number of clusters at 90% sequence identity to the total size of the family. Compared to the overall diversity score of 0.7, both TPP and Cobalamin classes had above-average diversities (0.71 and 0.82, respectively). However, these diverse classes did not pose any problems for the deep models. On the contrary, the AdoCbl and AdoCbl variant riboswitch classes posed significant learning challenges to both base and deep models. AdoCbl in particular is the smallest riboswitch family considered here, but is also unnaturally diverse (with a score of 0.83). This frustrates learning, because the limited number of instances do not adequately represent the class diversity, and result in class outliers. Consequently this emerged as the most challenging for all classifiers, reflected in the classwise F-scores (Supplementary Table S2). Four of the base classifiers managed a zero F-score, while the CNN and RNN achieved F-scores of 0.38 and 0.67, respectively, by far their lowest for any class. Even here, the consistency of the RNN model is remarkable. The other classes that were notably challenging to the base models but not to the deep models included Cyclic di-GMP-II, ZMP/ZTP, SAM 1–4 variant, Molybdenum co-factor, Glucosamine-6-phosphate and Guanidine-I riboswitch classes. Of these, the Glucosamine-6-phosphate, Cyclic di-GMP-II, and Molybdenum co-factor riboswitch classes are among the most diverse riboswitch families, with diversity scores ≥0.80. It is seen that the classification problems arise either with diverse classes or at the extremes of class sizes. Too large the class, the diversity is challenging, whereas too small and the learning itself is incomplete and challenged. The deep models – RNN and CNN – consistently performed well across all classes, independent of the size of the class. It could be inferred in this case that using direct features (i.e., sequences) rather than engineered features (i.e., k-mer frequencies) led to more robust models. From Table 5, it is also clear that most of the learnt classes (especially the large ones) are diverse, thereby elevating the classifier performance to robustness against adversarial attacks – that is, changing a few nucleotides in the input sequence would be unlikely to drastically alter the class prediction.

**TABLE 5:** Clustering analysis of riboswitch families at 90% sequence identity.

Rfam ID	^#^Clusters at 90%	Diversity	^#^Singleton clusters	^#^Redundant sequences	^#^Clusters in the rest
RF00050	2484	0.61	1866	46	45208
RF00059	8954	0.71	7366	272	38738
RF00080	611	0.73	493	29	47081
RF00162	3736	0.62	2722	141	43956
RF00167	2122	0.81	1818	59	45570
RF00168	1905	0.85	1707	45	45787
RF00174	11620	0.82	10130	289	36077
RF00234	814	0.87	731	11	46878
RF00379	2583	0.66	2044	97	45109
RF00380	441	0.42	265	35	47251
RF00442	600	0.67	449	46	47092
RF00504	3010	0.66	2345	66	44682
RF00521	495	0.61	379	20	47197
RF00522	214	0.40	113	28	47478
RF00634	501	0.40	317	12	47191
RF01055	978	0.80	865	73	46714
RF01057	629	0.76	519	13	47063
RF01482	150	0.83	132	12	47546
RF01689	131	0.62	101	2	47562
RF01725	435	0.55	326	25	47257
RF01727	149	0.62	105	16	47543
RF01734	1616	0.80	1389	127	46076
RF01739	344	0.31	215	56	47348
RF01750	1074	0.64	821	94	46618
RF01767	122	0.63	88	12	47570
RF01786	555	0.84	480	49	47137
RF01831	450	0.68	327	57	47242
RF02683	159	0.68	122	31	47533
RF03057	359	0.64	266	70	47333
RF03058	97	0.28	57	17	47595
RF03071	86	0.33	48	29	47606
RF03072	273	0.37	141	72	47419
ALL	47686	0.70	38871	1951	N-

*Singleton clusters indicate clusters with only one representative sequence. Redundant sequences are 100% identical to another member in the riboswitch family. The full set of riboswitch sequences (ALL) and ALL minus the riboswitch family of interest were also clustered.*

These results might be put in perspective by benchmarking against the existing literature. Guillen-Ramirez and Martinez-Perez (Guillén-Ramírez and Martínez-Pérez, 2018) extended the k-mer features logic and arrived at an optimal combination of 5460 k-mer features. Using a limited dataset of 16 classes, they used state-of-the-art machine learning to achieve accuracies in the high nineties, however, their results did not generalize equally to riboswitches with remote homology. For e.g., their best-performing classifier (Multi-layer Perceptron) misclassified 6 out of the 225 instances of Lysine riboswitch as cobalamin-gated. The source code for the features and modeling used in their work is not readily available for new applications. To make the workflow described in our study easily reproducible and user-friendly, we have developed a Python package riboflow^5^ mirroring the best RNN model. In addition to predicting the most probable class of a given riboswitch sequence, riboflow provides an option to predict the complete vector of class probabilities, which could be helpful in disambiguating any class confusion. It would also inform the design of synthetic orthogonal riboswitches for biotechnology applications. The implementation and usage details are provided in Appendix II (Supplementary File S1).

An interesting benchmark is afforded by the Riboswitch Scanner (Mukherjee and Sengupta, 2016), which used profile HMMs (Eddy, 2011) of riboswitch classes to detect riboswitches in genomic sequences. While our method addresses inter-class discrimination of riboswitch sequences, Riboswitch Scanner is a web-server that essentially performs riboswitch vs. not-riboswitch discrimination for user-given riboswitch classes. The absence of F-score metrics does not allow for direct comparisons, however, the sensitivity and specificity seemed consistently comparable for most classes, with noticeable variations with respect to the Glycine, THF and SAM I-IV variant riboswitch classes. It must be indicated that their method is validated with Rfam seed sequences, without consideration for the proliferation of riboswitch sequences. Performance evaluation on limited data could inflate performance estimates and complicate their interpretation. We further note that it is possible to extend our method to the ‘riboswitch-or-not’ classification problem by calibrating the prediction probability thresholds. In any case, our work enables the ranking of riboswitches using the strength of the predicted probabilities, which would aid the selection of the best riboswitch sequence design.

It must be noted that riboswitches are precisely specific to cognate ligands. For e.g., the AdoCbl riboswitch would not tolerate a methyl-substituted cobalamin (Nahvi, 2004) nor does the TPP riboswitch interact with thiamine or thiamine monophosphate (Lang et al., 2007). At the same time, these two riboswitches are very diverse in their phylogenomic distribution and actual sequences. The key to effective learning lies in treading a fine line between the intra-class diversity and inter-class specificity. It is remarkable the bidirectional LSTM RNN was able to achieve exactly this tradeoff. The roots of such performance of the deep models in general has been explained recently to be related to the lottery ticket hypothesis (Frankle and Carbin, 2019) as well as learning the intrinsic dimension of the problem (Li et al., 2018), here the classification of riboswitches.

To extend the functionality of our work, we have introduced a dynamic component to all our models, both base and deep. With the exponential growth in genome sequencing, the room for riboswitch discovery is enormous (Yu and Breaker, 2020). Our models could accommodate new riboswitch class definitions by way of dataset augmentation, thereby making them general and more robust. This work used the dynamic functionality to extend a preliminary 24-class model to the present 32 classes with sustained performance. The implementation and usage details of the dynamic functionality and other utilities provided in the repository are given in Appendix II (Supplementary File S1). It is noted that the deep learning models could be adapted to new classes and related problems by the technique of transfer learning (Weiss et al., 2016). Addition of new data to existing models presents data quality issues, which remain contentious (Leonelli, 2019), and could be partially addressed using the tools employed in this study to assess the canonical Rfam dataset.

In summary, we present riboflow, a python package (see foot note 5) as well as standalone suite of tools, that have been validated and thoroughly tested on 32 riboswitch classes. By using large and complete datasets, the variance of our modeling procedure has been optimized, and this ensures the generality and applicability of our models on new instances/classes without compromise of performance. riboflow is an off-the-shelf solution that is ready for programmatic incorporation of the RNN model into automatic annotation and design pipelines. All of our trained models are available to the interested user as ‘pickled’ models from https://github.com/RiboswitchClassifier. Riboswitches are a cornerstone of progress in synthetic biology, representing key checkpoints for translation activation. Our work presents an intuitive general-purpose extensible platform for the effortless characterization of new riboswitch sequences and classes, which would accelerate bacterial genome annotation, synthetic biology, and biotechnology, including the rapid design of novel genetic circuits with exquisite specificity. The predicted probabilities of class membership could be used as a proxy of aptamer binding strength with cognate signaling molecule, and this paves the way for the design of effective riboswitches for any stimulus or set of stimuli. In addition to being indispensable workhorses in synthetic biology, riboswitches represent novel and exciting targets for the development of new class of antibiotics (Penchovsky et al., 2020), and our work would also help toward the design of riboswitch inhibitors to combat emerging and multi-drug resistant pathogens. In addition, our work opens up the applications of deep learning methods, including advanced relatives like stacked Bi-LSTM and attention models (Vaswani et al., 2017), for addressing related and unrelated problems in the biological realm.

## Conclusion

We have demonstrated that CNN and RNN, without needing prior feature extraction, are capable of robust multi-class learning of ligand specificity from riboswitch sequence, with the RNN posting an F-score of ∼0.96. The confidence of classification could be obtained from an inspection of the predicted classwise probabilities. The bidirectional LSTM RNN model has been packaged into riboflow to enable embedding into genome annotation pipelines, genetic circuit-design automation, and biotechnology workflows. The CNN shows the best tradeoff between the time-cost of training the model and overall performance and could be applied to the task of learning new riboswitch classes using the provided dynamic update option that is provided. All the code used in our study is freely available for any use and further improvement by the scientific community as well as in the larger interest of reproducible research. Our study has highlighted the use of macro-averaged F-score as a disciminating objective metric of classifier performance on multi-class data. Our work reaffirms the competitive advantages of bidirectional LSTM RNNs over conventional machine learning and hidden markov profiles in modeling data sequences, and opens up their applications for modeling other non-coding RNA elements.

## Data Availability Statement

All datasets generated for this study are included in the article/Supplementary Material.

## Author Contributions

AP conceived and designed the work and wrote the manuscript. KP, RB, and AP performed the experiments and analyzed the data. All authors contributed to the article and approved the submitted version.

## Conflict of Interest

The authors declare that the research was conducted in the absence of any commercial or financial relationships that could be construed as a potential conflict of interest.

## Footnotes

1 https://github.com/RiboswitchClassifier

2 www.sklearn.com

3 http://keras.io

4 https://github.com/RiboswitchClassifier in the Datasets folder.

5 https://pypi.org/project/riboflow/
