# Supplementary material for: Riboflow: Using Deep Learning to Classify Riboswitches With ∼99% Accuracy
Source: Front Bioeng Biotechnol. 2020 Jul 14;8:808. doi: 10.3389/fbioe.2020.00808 (PMC7371854; doi:10.3389/fbioe.2020.00808)
Supplement: Supplementary file 1 [file Data_Sheet_1.ZIP › File S1.pdf]

Supplementary Information

Appendix I. Hyperparameter tuning

Model hyperparameters impact the model performance, and the model may be parameterised with the optimal hyperparamters for the best performance on the test set. Hyperparameter optimization is usually neglected in most biological data mining studies. Here we provide a discussion of the hyperparameter optimization carried out in our study for both base and deep machine learning models.

Base machine learning models:
Each model is parameterised with a default set of its values, which may not be suitable for the problem at hand. Hyperparameter tuning was performed by scanning a grid of possible values for each parameter. This approach is called Grid-Search, and it entails a combinatorial model search. We used 10-fold internal cross validation (i.e, on the training set) to optimize the hyperparameters. The test set was not used in hyperparameter optimization, but the effectiveness of the optimization process could be captured by comparing the performance of the model **before and after** hyperparameter optimization.

Table S1. Hyperparameter optimization of the base models. The set of **o**ptimal hyperparameter values following a grid search yielded better model accuracies on the test set relative to test set accuracy with the default settings and prior to optimization.

Model	Hyperparameter	Grid search	Optimal value	Test-set accuracy **before, after**
Random Forest	Number of estimators	1000, 2000, 3000, 4000, 5000	3000	0.63, 0.70
	Max depth	60,70,80,90,100	80	
	Criterion	Gini,Entropy	Gini	
Decision Tree	Maximum features	Auto, Sqrt, log2,None	None	0.48, 0.53
	Minimum sample split	2,3,4,5,6,7,8,9, 10,11,12,13,14, 15	3	
	Minimum sample leaf	1,2,3,4,5,6,7,8, 9,10,11	8	
	Random state	123,345,None	None	
	Max depth	5,10,15,20,25, None	15	
KNN	Number of neighbours	5,6,7,8,9,10	8	0.60, 0.65
	Leaf size	1,2,3,5	1	

	Weights	uniform, distance	distance	
	Algorithm	auto, ball_tree, kd_tree, brute	auto	
AdaBoost	Number of estimators	1, 50, 1000, 2000, 3000	1000	0.24, 0.44
	Learning rate	0.01,0.1,1.0,5.0, 10.0	1.0	
	Algorithm	SAMME,SAMME.R	SAMME	
Multi Layer Perceptron	Activation	Tanh, Relu	Relu	0.68, 0.72
	Solver	SGD, ADAM	ADAM	
	Alpha	0.0001, 0.01, 0.1, 0.05, 1.0	0.01	
	Learning rate	Constant, Adaptive	Adaptive	

In the case of the multi-layer perceptron, the default number of hidden layers (=1) with the default number of nodes (=100) was retained, since changing these did not appear to affect the performance. Even with the heuristic scan of hyperparameters, it is seen that there is a marked improvement in performance of all models, underscoring the importance of hyperparameter optimisation. A model-wise exhaustive scanning of hyperparameters would be costly in time and resource and unlikely to further improve performance.

Deep learning models:
For the DL models, the hyperparameter space is so large that it is not practical to execute a heuristic scan of hyperparamters. Instead, we took a train-validate-test approach with a 0.8:0.1:0.1 split, effected by setting the 'validation' flag in keras to 0.1 (see Methods section). Since the error difference between the training and validation sets was insignificant, and the DL models performed exceedingly well on the classification task, the default / initial values of the model parameters were left unchanged for both the RNN and CNN. These parameters are recapitulated below:

CNN
Objective function: accuracy (scorer)
Loss function: error i.e, 1 - accuracy
Filters = 10
kernal size = 3
Conv1D layers = 2
Epochs = 20
Optimiser = RMSPROP
Activation = Relu
maxPooling

Dropout = 0.5
Batch size: 128.

RNN
Objective function: accuracy (scorer)
Loss function: error i.e, 1 - accuracy
Bidirectional layers = 2
Epochs = 25
LSTM nodes = 62
Optimiser = ADAM
Activation = Relu
Dropout = 0.2
Batch size: 128.

Our experiments in varying parameter values indicated that the CNN performance tended to saturate with test-set accuracies of 97 - 98%. Similarly, the RNN performance tended to saturate with test-set accuracies of 99%. For references on systematic hyperparameter optimisation for deep learning networks, the interested reader is referred to 'Neural Architecture Search' (for e.g, see: Elsken, Thomas; Metzen, Jan Hendrik; Hutter, Frank (August 8, 2019). "Neural Architecture Search: A Survey". Journal of Machine Learning Research. 20 (55): 1–21).

Appendix II. Implementation and Usage details.

`riboflow` usage:
The Python package `riboflow` (https://pypi.org/project/riboflow) could be installed using the Python package installer, `pip (or pip3)`, and the dependencies `numpy, tensorflow` and `keras`. The following interactive code gives an example of `riboflow` uses.

```
> import riboflow

#Construct a Python list of riboswitch sequences. A sequence is a string in
alphabet 'ATGC'
> sequences = [
"TTTTTTTTGCAGGGGTGGCTTTAGGGCCTGAGAAGATACCCATTGAACCTGACCTGGCTAAAACCAGGGTAGGGAATTGC",
"CTCTTATCCAGAGCGGTAGAGGGACTGGCCCTTTGAAGCCCAGCAACCTACACTTTTTGTTGTAAGGTGCTAACCTGAGC",
"CCACGATAAAGGTAAACCCTGAGTGATCAGGGGGCGCAAAGTGTAGGATCTCAGCTCAAGTCATCTCCAGATAAGAAATA"
]

#Use case 1. Return the most probable class for each riboswitch sequence:
> riboflow.predict(sequences, "predict_class")

#Use case 2. Return the complete vector of class probabilities for each riboswitch
sequence, to disambiguate potential class confusion:
> riboflow.predict(sequences, "predict_prob")
```

Dynamic functionality:
The script `dynamic.py` (https://github.com/RiboswitchClassifier) implements the dynamic functionality and has two use cases depending on the number of new classes.

```
#Use case 1: Only one new class.
$ dynamic.py -fa riboclass.fa
```

```
#Use case 2: Multiple new classes.
$ dynamic.py -d dir
```

In the simpler use case, a single new class is added to the dataset by specifying the option '-fa' and providing the name of the fasta datafile. In the second use case, multiple new class definitions could be added to the model by specifying the '-d' option and providing the name of the directory that contains the fasta datafiles for the classes, one for each new class. The script pre-processes the datafiles for compatibility and the alphabet, and adds them to the existing dataset. Essentially it is used to generate the equivalent processed csv datafiles with the sequence and the k-mer (i.e, mono- and di-nucleotide) frequencies. These files represent the updated datasets, which could be directly read as input for training the new base models, and the CNN and RNN deep models. These files are used for training with baseModels.py as well as the deep learning applications, rnnApp.py, and cnnApp.py, described below. The new models would then be able to handle any number of new riboswitch classes, regardless of sequence diversity considerations. The performance of the deep models remained unaffected by the number of classes used for learning, and the models could be expected remain robust as more riboswitch classes are discovered.

Training the CNN on an Intel i7 processor @3.4GHz with 8GB RAM took ~ 8 minutes, whereas training the RNN took about 5 hours. In the event of many new riboswitch classes, our recommendation would be to dynamically update the CNN model (`cnnApp.py`) available at https://github.com/RiboswitchClassifier. The trained deep models are available in hdf5 format in the same repository, and are modest in size (CNN model: 233 Kb; RNN model: 1.8 Mb).

Other utilities in the Repository:

1. original_datasets

```
a. 32_riboswitches_fasta    --> Fasta   Format of the 32 riboswitches
b. 32_riboswitches_new_csv  --> CSV     Format of the 32 riboswitches
```

2. processed_datasets

```
a. final_32classes.csv                --> Original 32 riboswitches Dataset cleaned
and Frequencies calculated
b. final_32train.csv          --> 90% of each riboswitches label in the
final_32classes.csv
c. final_32test.csv           --> Remaining 10% of each riboswitches label in the
final_32classes.csv
```

3. models

```
Contains the rnn and cnn model's in h5 format
```

4. preprocess.py

```
Contains various utilities for train:test splitting of the dataset, loading the
datasets and other preprocessing of the data. Could be used to generate -mer
frequencies, final_train.csv, final_test.csv and used for data preprocessing by all
the models (i.e, both base and deep learning models: baseModels.py, rnnApp.py and
cnnApp.py)
```

5. multiclassROC.py

Used for the ROC analysis of all models (i.e, base and the deep learning models).

sklearn Base Models

> python3 baseModels.py

1. Create's a Picked Model for each of the sklearn classifers stated below:
    AdaBoostClassifier(),
    GaussianNB(),
    KNeighborsClassifier(),
    DecisionTreeClassifier(),
    RandomForestClassifier(),
    MLPClassifier()
2. Each model is used on the test set to obtain accuracy, generate a classication report and the ROC-AUC values for each
   of the 32 classes.
3. The MLPClassifier() proved to be the best among the chosen sklearn classifiers and hence Neural Networks (CNN and RNN)
   were explored further to acheive greater accuracy.

keras.tf RNN

> python3 rnnApp.py

1. Creates a .h5 RNN Model using tensorflow on keras
2. The model is used on the test set to obtain accuracy, generate a classication report and the ROC-AUC values for each
   of the 32 classes.
3. Provides an Accuracy of 99% on the test set.
4. New layers and hyperparameter values can be added or changed when dealing with a dataset having different number of classes
5. The train time is fairly long (in the magnitude of hours - suitable for system with high specs)

keras.tf CNN

> python3 cnnApp.py

1. Creates a .h5 CNN Model using tensorflow on keras
2. The model is used on the test set to obtain accuracy, generate a classication report and the ROC-AUC values for each
   of the 32 classes.
3. Provides an Accuracy of 97% on the test set.
4. New layers and hyperparameter values can be added or changed when dealing with a dataset having different number of classes.
5. The train time is fairly short (< 1 min - suitable for low spec systems)
